# Exploring the Influence of Public Perception of Mass Media Usage and Attitudes towards Mass Media News on Altruistic Behavior

**DOI:** 10.3390/bs13080621

**Published:** 2023-07-26

**Authors:** Chi-Horng Liao

**Affiliations:** 1 Department of Communication Studies, Tzu Chi University, Hualien 97004, Taiwan; lchjerry@mail.tcu.edu.tw; 2 Bachelor Program in Digital Media and Technology, Tzu Chi University, Hualien 97004, Taiwan; 3 Media Production and Education Center, Tzu Chi University, Hualien 97004, Taiwan

**Keywords:** mass media exposure, perception, altruistic behaviors, credibility, social influence, the theory of planned behavior (TPB), structural equation modeling (SEM), cultivation theory, Tzu Chi Foundation

## Abstract

Based on the cultivation theory and the theory of planned behavior, this study determined how people’s perceptions of mass media news and their attitudes towards it impact their altruistic behavior by examining the factors that influence perception. The study collected data from 435 individuals with access to mass media in Taiwan, which were analyzed using SEM. The results revealed that media exposure, credibility, and social influence were critical factors that influenced individuals’ perceptions of mass media news, with media exposure having a more significant influence. Surprisingly, the findings demonstrated that perception was negatively related to attitudes, inconsistent with the proposed hypothesis. Thus, perceptions and attitudes were positively associated with altruistic behavior, and attitude was found to mediate the relationship between perceptions about mass media news and altruistic behavior. The study also provides important implications for theory and practice, especially in mass media entities, in developing and adopting practices that promote trust among the audience by encouraging altruistic behaviors through news coverage of various issues.

## 1. Introduction

There are numerous problems that the public needs to address in the modern world. These global problems include spreading pandemics and other diseases, refugee crises due to political conflicts, and natural disasters, which render most people homeless and in need of different kinds of support. To ease the impact of these issues, people must engage in altruistic acts such as providing medical aid to the displaced, integrating refugees, and making donations [1]. In the modern world, helping one another to bring about positive social outcomes for the public good is the purpose of many organizations. Therefore, on the road to achieving this purpose and goal, organizations must search for donors who can provide different resources in terms of money, time, physical effort, and other contributions from the general public. The success of these organizations in helping others relies on the extent to which they can attract such donors. To raise funds for altruistic causes, organizations must understand public giving behavior. Altruistic behavior is beneficial to individuals and society at large. For example, it can ensure the well-being of individuals both mentally and physically [2]. Additionally, it holds considerable significance for humanity’s collaborative efforts and societal advancements. Hence, the factors influencing donors’ altruistic behavior must be understood and analyzed. 

Achieving altruistic behavior among the audience depends on whether they perceive the news from mass media positively or negatively. The fact that the audience demonstrates behavior that either supports or does not support altruism depends on their perceptions and attitudes toward the positive and negative impacts of mass media news [3]. It is essential to investigate the factors that influence the perception of the public towards mass media news and how they relate to attitudes toward mass media news and altruistic behavior. Perceptions and attitudes influence how people behave. 

Perceptions, attitudes, and behaviors have been linked in several past studies. For example, there have been observable relationships between user perceptions, attitudes, and behavior toward social media advertising in three countries (South Africa, Australia, and Germany) [4]. The existence of the relationships between perception, attitudes, and behavior regarding social media advertisements was empirically demonstrated in the study. Research has also examined the online factors influencing consumers’ perceptions and attitudes toward advertising on social media. The findings revealed a significant positive relationship between perceived interactivity and attitude toward social media advertisements and a positive relationship between credibility and attitude toward these advertisements [5,6].

Another study also investigated the youth market’s perceptions, attitudes, and behavior towards internet banking services [7]. It was discovered that young individuals, aged 16 to 29, had higher positive opinions and behavioral intentions toward online banking services than the other user groups in their study. This study also demonstrated the presence of linkages between perceptions, attitudes, and behavior. Likewise, another study discovered that residents’ perceptions of tourism’s positive economic and cultural impacts influence not only their attitudes toward tourism, but also their attitudes toward tourists. The study aimed to explain how the residents’ perceptions of tourism’s impacts on host communities influenced not only their attitudes toward tourism, but also their attitudes toward tourists. They found that both types of attitudes influence behavioral support for tourism [8]. 

Thus, these studies have demonstrated the relationships among perceptions, attitudes, and behavior, and have proven the existence of the relationships among the variables. However, as observed from the above-mentioned previous studies, most of the focus is on consumer behavior, and there is still a need for more information on the public’s giving behavior. Secondly, previous studies have overlooked factors that could influence consumer perceptions. Our study contributed to the literature by developing a model of public perceptions and attitudes that incorporates how the public’s perception of mass media news is brought about. This model suggests the ways these perceptions could be enhanced, as they are crucial in influencing people’s attitudes towards mass media news, which influences their engagement in altruistic behavior. Thus, this study investigates how different factors indirectly influence altruistic behavior through perceptions and attitudes, making it one of the few studies to examine this meaningful relationship. The study also adds to the existing literature on altruism (or altruistic behavior) and how it relates to perception and attitude. Thus, this study’s primary aim was to identify the various elements that shape public perception of mass media news. The current study employs the cultivation theory and the theory of planned behavior (TPB).

The study employs a cross-sectional study design in which data were collected from the general public in Taiwan exposed to mass media news through a questionnaire survey using a convenient, nonprobability sampling method. Structural equation modelling (SEM) was the primary analytical tool used to test this study’s hypotheses. The Analysis of Moment Structures (AMOS) Covariance-Based Structural Equation Modelling (CB-SEM) software was used.

### 1.1. Study Objectives

The study’s main objective was to investigate the factors affecting public perceptions of the use of mass media news concerning the environment, disasters, global aging, strange diseases/pandemics, and poverty-related information, and how these perceptions shape their attitudes toward altruistic behaviors. The specific objectives of the study include examining the relationship between exposure to mass media and people’s perceptions of mass media news, examining the relationship between the credibility of mass media news and people’s perceptions of mass media use, examining the relationship between social influence and people’s perceptions about mass media news/message, investigating the direct relationship between people’s perceptions about mass media news/message and altruistic behavior, investigating the relationship between people’s perceptions about mass media news/message and attitudes toward mass media news, and finally establishing whether attitudes toward mass media news mediate the relationship between publics’ perceptions about the use of mass media and altruistic behaviors. 

### 1.2. Research Structure

This paper comprises eight sections. Section 1 serves as the introduction, providing the study’s background, research motivation, objectives, research gap, and overall structure. Section 2 presents a literature review, exploring relationships among constructs and proposing research hypotheses. Section 3 outlines the study’s methodology, encompassing the research design, measurement items of constructs, data collection method, and data analysis approach. This section also addresses achievement validity and reliability. Section 4 presents the formal survey and the subsequent data analysis. Section 5 discusses the study results and their correlation with previous studies and theories. Section 6 delves into the theoretical underpinnings of the current study’s findings, along with their practical implications. In Section 7, the author acknowledges the research limitations and offers suggestions for future studies. Finally, the concluding section summarizes the results.

## 2. Literature Review and Hypotheses Development

This section provides an overview of the subject under study and the supporting theories. It outlines the key concepts of our theoretical model, encompassing aspects like media exposure, credibility, social influence, perceptions about mass media news, attitudes toward it, and altruistic behavior. Additionally, we propose potential connections between these variables and develop hypotheses to be examined in our research.

### 2.1. Cultivation Theory

The study applied the cultivation theory to elucidate the relationships among the constructs. According to the theory, individuals consistently exposed to media content over a prolonged period tend to interpret social realities based on how they are portrayed. Consequently, this exposure has an impact on the attitudes and behaviors of the audience [9]. The theory specifically addresses how regular television program viewing can shape people’s understanding of reality, implying that mass media significantly influences their perception of the world. According to the cultivation theory in mass communication, excessive consumption of media content that disproportionately emphasizes positive or negative themes or news can adversely affect the audience [10]. In other words, exposure to skewed content may negatively affect individuals’ perceptions and attitudes [10]. 

### 2.2. The Theory of Planned Behavior

The theory of planned behavior (TPB) has been employed to establish the relationship between certain independent variables and the dependent variable of altruistic behavior. This theory offers a practical conceptual framework for understanding the complexities of human social behavior [11]. According to the TPB, an individual must first intend to perform a certain action before actually doing it. This intention is determined by their motivation, collective expectations, and perception of the ease or difficulty of executing the behavior [12]. Furthermore, the TPB predicts that when individuals express their intention to adopt a particular behavior, it is likely to be manifested in their actions, particularly in situations where they have sufficient control over their behavior [13]. 

The mass media plays a significant role in providing the public with information and awareness about emerging issues and novel concerns [14]. Media coverage of lesser-known issues can impact the level of public concern for these matters, leading to increased attention from the public [2]. Furthermore, the media can influence the problems that the public perceives as important. The way news is presented can shape how readers or viewers perceive and form specific values and opinions. According to the TPB, personal actions are driven by intended behavior, which in turn is influenced by attitudes (one’s desires), subjective norms (the opinions of others), and perceived behavioral control (one’s belief in their ability to act) [15]. News consumption is equivalent to consuming information, ultimately impacting people’s perspectives [16]. The TPB also suggests that one’s attitude can be used to predict possible actions. If an individual holds a more favorable attitude towards a particular behavior, their intention to engage in that behavior will be greater. Conversely, if their attitude toward a behavior is negative, their inclination to engage in it will be diminished. So, it is the attitudes toward mass media news that influence the altruistic behavior of the public. 

### 2.3. Media Exposure and Public’s Perceptions about Mass Media News/Message

Media exposure and usage have a significant impact on shaping views, attitudes, and behaviors among media users. The frequency of communications or media content to which individuals are exposed and the extent to which they retain that information is referred to as media exposure [17]. Media plays a crucial role in influencing people’s perceptions and behaviors by disseminating information, raising awareness, and providing education. It facilitates communication among individuals and enables them to gain insights into various global, social, and environmental concerns [18]. 

Moreover, media exposure fosters feelings of promotion and generates perspectives that foster altruistic behavior. A study revealed that the internet, television, and newspapers are the most commonly utilized channels for obtaining information about international topics such as climate change, natural disasters, and pandemics. Media coverage emerged as the most influential source of knowledge concerning these subjects [19]. The media to which people are exposed significantly shapes their beliefs, opinions, and actions, substantially impacting their comprehension of altruism-related issues. 

Research has consistently shown that an individual’s perceptions can be influenced by the extent of their exposure to a particular subject. For instance, a study revealed that exposure to mass media has a positive impact on an individual’s perception of personal responsibility towards the environment, subsequently affecting their behaviors in relation to environmental concerns [20]. The research investigated the influence of media exposure and engagement with social networking sites on environmental concerns. Similarly, individuals’ positive evaluations of particular television programs stimulate their interest in altruistic behaviors. For example, witnessing heroic deeds and empathizing with prosocial behavior can inspire individuals to model such conduct [21]. Exposure to messages related to altruism through mass media coverage also plays a role in shaping people’s awareness and concerns regarding generous or helping behaviors. As the cultivation theory supports, frequent exposure to media content can influence individuals’ perceptions and interactions with their environment, ultimately affecting their behaviors [9]. Consequently, extensive mass media coverage can increase the public’s perception of giving or helping behavior. Based on this understanding, the study hypothesized that:

**Hypothesis** **1 (H1).** *Exposure to mass media positively relates to the increased positive perception of the mass media news/message*.

### 2.4. Credibility and Public’s Perception of Mass Media News 

Credibility in media news/messages refers to the extent to the degree which receivers perceive the information as trustworthy and believable [8]. Alternatively, media credibility is defined as a perceived quality based on various factors, including trustworthiness and expertise [22]. In marketing, credibility pertains to the believability of product information within a brand, necessitating consumers’ perceptions that the brands possess the ability, expertise, willingness, or trustworthiness to consistently deliver what has been promised [23]. Media credibility has been explored within two major domains: source and medium. Source credibility has been investigated in interpersonal, organizational, and mass-mediated situations by examining how different communicator traits might impact message processing. A communicator can be an individual, group, or organization. On the other hand, medium credibility focuses on the channel through which material is provided rather than the source of the content [24].

How people view the media significantly influences their behaviors. The general public, government entities, organizations, and advocacy groups hold different media perspectives. The public’s perception of the veracity and accuracy of the information in the media is said to influence the initiation of legislation and discussions on various issues [25]. While scholars have long debated the credibility of the source and message, message acceptance behaviors are directly influenced by how people perceive the medium. When the medium, source, and information are perceived as accurate, people tend to rely on the information and become persuadable [26]. Therefore, people base their opinions on altruism on the veracity, dependability, and accuracy of the media sources and material to which they are exposed. Thus, media credibility is a crucial concept, especially considering the influence of mass media news on global issues. The credibility of the source impacts a person’s willingness to change perceptions based on the information provided. 

Several empirical studies have demonstrated a clear relationship between media credibility and perception. For instance, research has shown that the credibility of an eWOM (Electronic word of mouth) source, as indicated by dimensions of expertness and trustworthiness, positively impacts risk perception [27]. This implies that the media, through which a message is conveyed, directly has an influence on its believability. Credibility is most significantly influenced by factors such as trustworthiness and knowledge, with the reliability of a source contributing to its perceived morality or goodness. 

Nevertheless, expertise is primarily defined by knowledge, experience, and competencies [28]. These findings show that media news with high credibility is perceived as possessing high levels of trustworthiness and expertness. Conversely, information from unreliable sources requires more scrutiny and elaboration than compared to highly trustworthy sources [29,30]. Individuals become more confident of the data since they perceive the source positively. Therefore, based on the above argument, this research proposes that:

**Hypothesis** **2 (H2).** *Credibility (trustworthiness and expertness) of the mass media positively relates to the public’s positive perception of the mass media news.*

### 2.5. Social Influence and Public’s Perception of Mass Media News

Social influence refers to the changes generated in an individual’s mindset due to external sources, such as communicated information [7]. It involves the individual’s belief about how significant others perceive whether they should engage in a particular behavior, known as social influence [31]. The exploration of social impact seeks to comprehend how external factors, like communicated data, can alter an individual’s perceptions and attitudes [7]. Social influence could be a driving force for individuals exposed to mass media news/messages, as it influences their perception of its usefulness. Mass media and internet websites can act as sources of social influence, as individuals seek information from various sources to learn about different issues [15]. Social influence leads people to adjust their attitudes, behaviors, opinions, or beliefs to align with those of the majority, a phenomenon known as conformity [32]. Studies have demonstrated that social influence relates to youth risk perceptions [33]. Hence, the study proposed that:

**Hypothesis** **3 (H3).** *Social influence positively relates to public perceptions of mass media news.*

### 2.6. Perceptions of People about Mass Media News/Messages and Altruistic Behavior

Inherently, humans are social, and many of their thoughts, decisions, and behaviors revolve around their interactions with others. A social phenomenon that garnered significant attention is altruism [34]. Altruism is defined as a selfless act performed for the benefit of another. This behavior seeks to promote the welfare of others without any conscious regard for one’s self-interests [21]. Humans demonstrate and act upon this genuine concern for the well-being of others. Understanding the factors that influence altruistic behavior has been the subject of extensive research in psychology and economics for many decades [1]. However, prior research in these two fields on altruistic behavior has mostly been conducted independently. Therefore, this study introduces the concept of “altruistic behavior,” which describes the motivational state of individuals who actively promote the well-being of others at their own expense [25]. Altruism can be understood in terms of two main concepts: (1) behavioral altruism, which refers to observable actions that benefit others, and (2) psychological altruism, which involves the underlying motivations and intentions behind such behavior.

Regarding behavior, altruism encompasses any action taken out of altruistic intentions. From a psychological perspective, altruism involves the willingness to sacrifice one’s welfare to benefit someone else [29]. Despite many definitions of altruism, they all share a common theme, implying an act of benevolence carried out for the good of others, with no thought of personal gain or detriment. Altruistic behavior is described as voluntary action undertaken to help others without expecting external rewards, compensation, or to avoid punishment [3,35]. Most authors agree that actual altruistic behaviors are acts of kindness performed to help others, driven by one’s own free will, with the sole purpose of benefiting the other person, and without expecting anything in return. The focus is on benefiting others, though it may only sometimes be beneficial.

Altruistic behavior is related to prosocial behavior, as the authors have established similarities and differences between the two. For example, both behaviors share a similar focus on advancing the well-being of others besides the individual carrying out the action [14]. In other words, both behaviors involve helping people in need without expecting anything in return. However, the two differ in their intents and motives, costs and benefits, and social context. Altruism refers to the drive to improve the well-being of others as the ultimate objective, while prosocial behavior encompasses a broader range of deeds performed to benefit individuals or groups other than oneself [36]. In terms of costs and benefits, altruism is characterized as a behavior that incurs costs for the individual performing it but yields benefits for the recipient.

In contrast, prosocial conduct encompasses any action that benefits someone else. To elucidate the distinction between prosocial behavior and altruism in a social context, it is worth noting that the former involves actions highly esteemed and anticipated by society. On the other hand, altruism is more precise, focusing on activities specifically aimed at advancing the well-being of a group as a whole [14].

A person’s level of altruism and its underlying causes can be traced back to their social learning background. Families, educational institutions, and the mass media play significant roles in socializing individuals in a given society. The mass media, in particular, can influence individuals by teaching acceptable behaviors through media messages [2,37]. Additionally, media coverage can inspire people to get involved in crisis relief initiatives during a natural or human-caused catastrophe [9]. The mass media constantly instruct people on acting, feeling, and thinking.

People’s perceptions of media have been found to significantly impact their actions [38]. Public opinion and stakeholders’ reactions to initiate legislation and engage in discussions are influenced by their perception of the credibility of the mass media news/messages [25]. When individuals perceive the media, its sources, and messages as trustworthy and reliable, they become convincing. Previous studies have primarily focused on how individuals’ behavior in areas such as politics is affected by their perception of media. However, this study concerns how people’s perceptions of mass media news/messages about altruism affect their altruistic behaviors. Therefore, this study suggested that:

**Hypothesis** **4 (H4).** *Perceptions of mass media news positively relate to altruistic behavior.*

### 2.7. Perceptions and Attitudes towards Mass Media News/Messages

To understand the effect of mass media news/messages on people’s selfless actions, it is necessary to investigate how the public responds to the message and gain insight into their attitudes. Attitude refers to a mental inclination demonstrated by appraising a particular item with some extent of approval or disapproval [39]. Gaining insight into the public’s attitude means understanding whether they favor or oppose media coverage regarding charitable behavior and whether they are open to adopting such behavior. People form their perceptions of mass media news/messages through exposure, social influence, and credibility of the source and message [8].

If the audience is consistently exposed to and finds the mass media news trustworthy, their attitudes toward the media’s coverage of global disasters and altruistic behaviors will be positively influenced. For instance, it has been argued that based on their perceptions of exposure, credibility, and social influence, the audience subsequently forms their attitudes toward news/messages from mass media about altruistic behavior [8]. Similarly, the correlation between individual perceptions and attitudes toward certain behaviors has been verified [40]. Thus, the authors of this study expected to explore whether the audience’s perception on the credibility and reliability of the news/message they are exposed to leads to a positive attitude toward altruistic behavior. Hence, in this regard, perceptions of the mass media news/message about the global crisis and altruistic behavior determine audience attitudes toward the mass media news/message. Furthermore, this research expects to find a mediation effect of the attitudes towards mass media news/messages in the direct relationship between perceptions of mass media news/messages and altruistic behavior, as perceptions are also related to attitudes towards mass media news/messages. Therefore, this study proposed the following hypotheses:

**Hypothesis** **5 (H5).** *Perceptions about mass media news/messages positively relate to attitudes toward mass media news/messages.*

**Hypothesis** **6 (H6).** *Attitudes toward mass media news/messages mediate the relationship between perceptions of mass media news/messages and altruistic behavior.*

### 2.8. People’s Attitudes toward Mass Media News/Messages and Altruistic Behavior

The theory of planned behavior (TPB) states that people’s behavior is mainly determined by their attitudes, which can be understood as how they view and evaluate an object [8]. The TPB has been employed to forecast the actions of people based on their attitudes. Researchers suggest that if the audience or people have a favorable view of mass media news/messages about altruism, they will be more likely to participate in such behaviors [41], such as participating in charitable activities and giving donations. The attitude of the public towards news/information disseminated by mass media will play a crucial role in determining their level of involvement in such altruistic activities. How people perceive, think about, and believe in the news/message from the mass media significantly impacts on whether or not they will participate in altruistic behavior. 

Previous research has determined an observable relationship between an audience’s attitude and willingness to be involved in aiding activities. Thus, strong attitudes are considered good predictors of behavior [41]. For example, a study measuring the altruism levels and attitudes of nursing students towards their profession demonstrated that the participants had a higher-than-average level of altruism and held positive feelings about nursing [34]. This indicates that the more positive the public’s outlook on mass media news/messages, the more likely they are to participate in assisting or donating activities. Consistent with the TBP, if someone has a more positive attitude toward a specific action induced by their perceptions, their willingness to act in that particular way will increase [13].

Conversely, if their attitude is negative, their willingness to act will decrease. As negativity increases, the intention to engage in the behavior will also decrease. Therefore, a positive attitude towards mass media news/messages results in increased engagement in altruistic behavior. Consequently, this research proposed that:

**Hypothesis** **7 (H7).** *People’s positive attitudes towards mass media news/messages positively relate to their altruistic behavior.*

**Figure 1:**
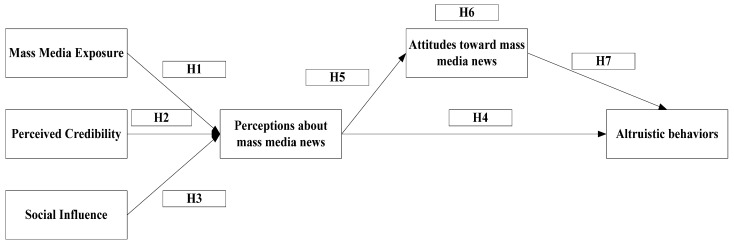
Research framework.

Figure 1 illustrates the proposed research framework for the study. The framework includes the primary constructs and their interrelationships, highlighting the key factors that are investigated to understand the influence of mass media news on altruistic behavior. The framework serves as a visual representation of the theoretical model that guides the empirical investigation in this study.

## 3. Research Methodology

### 3.1. Sampling Method and Procedure

The data for this study were collected using a questionnaire survey conducted through a convenient sampling method, which falls under the category of non-probability sampling. Convenient sampling is a commonly used form of sampling in population research. It was chosen for its popularity due to its cost-effectiveness, time efficiency, and simplicity. Convenience sampling involves collecting participant data because individuals are readily and easily available. It selects participants who are accessible in a specific location [42]. However, it is important to note that convenient sampling has some inherent weaknesses. One of the main drawbacks is selection bias, which occurs when certain individuals are more likely to be included in the sample than others, leading to a non-representative sample. As a result, the sample may not accurately reflect the characteristics of the more significant or general population being studied. 

This limitation can impact the external validity or generalizability of the study’s findings. Additionally, convenient sampling is not recommended for descriptive or causal research, as it may introduce biases and limit the ability to draw meaningful conclusions about cause-and-effect relationships. While it may provide quick and accessible data, researchers should be cautious about generalizing the results beyond the specific sample used in the study [43]. The potential risk of motivation bias in the study due to the motivation of participants is also an important point to consider. Motivation to participate may influence the willingness of individuals to be involved in the research, potentially affecting the representativeness of the sample. By recruiting more participants, the researchers aimed to enhance the diversity and representation within the sample, which can help to mitigate the impact of motivation bias. The decision to conduct the study in Taiwan is justified by its vulnerability to natural disasters, such as its location in the earthquake belt and being on major tracks of typhoons in the northwest Pacific area and the East Asia monsoon system [44]. Conducting the study in a region frequently affected by natural disasters is relevant and appropriate, as it allows for an exploration of the relationships between media exposure, attitudes, and altruistic behaviors in the context of global crises. By acknowledging the limitations of the sampling technique and providing a rationale for the choice of study location, the researchers carefully consider potential biases and context-specific factors. Researchers should continue to be aware of these limitations and take them into account when interpreting and generalizing the study’s results.

The study participants consisted of the general public, specifically those exposed to mass media news related to current global issues such as natural disasters, pandemics, other disease outbreaks, and environmental issues. The questionnaire used screening questions to identify these participants to confirm their media exposure. The questionnaires were created using google forms, an online survey tool, and were transferred to the participants electronically, including social networking sites, email, Instagram, and Messenger. Participation in the study was voluntary, and respondents willingly took part after being informed about the research purpose. A cover letter accompanying the questionnaire indicated that the survey was conducted solely for academic research. The participants were assured of the confidentiality of their responses, ensuring that their identities and data would remain anonymous and protected. To cater to the local language, the scales used in the study were written in Chinese. This decision was made to ensure that the participants were comfortable and could easily comprehend the questions and respond accurately. 

The researchers’ efforts in distributing 467 questionnaires and receiving 450 responses, with a high return rate of 96.4%, are commendable as it reflects a substantial representation of the target population. This robust response rate enhances the reliability and validity of the data collected, increasing confidence in the study’s findings and their applicability to the general public exposed to mass media news on global issues in Taiwan. The study’s well-structured and comprehensive approach to participant recruitment and data collection ensured that the research was conducted ethically and effectively. The high response rate further supports the credibility of the study’s findings.

The study was conducted over a period of ten weeks, during which 450 responses were collected from participants. After addressing missing values in the data, a total of 435 responses remained for analysis. The presence of missing data poses several challenges that necessitate careful handling [45]. Missing values can reduce statistical power, which refers to the probability of correctly rejecting the null hypothesis when it is false [46,47]. Additionally, missing data can lead to biased parameter estimations, limited sample representation, and increased data analysis complexity due to the need for additional information [46]. To address this issue, we employed a data deletion approach to remove records with missing values, ensuring a complete dataset for analysis.

Another important bias to consider is social desirability bias. Participants may have responded in a manner they perceived as socially desirable, particularly for constructs related to doing good to others, potentially influencing the results. Social desirability bias can impact mean scores, constrain the variability of responses, and affect correlations between variables, leading to inconsistent conclusions [48]. Researchers should be aware of this bias and take measures to minimize its influence, such as using anonymity in data collection or employing techniques to assess and control for social desirability bias in the analysis. By acknowledging these potential biases and limitations, the researchers demonstrate transparency and awareness of data collection and analysis challenges. It is essential for researchers to address and mitigate biases to ensure the accuracy and reliability of their study findings [49].

### 3.2. Questionaire Design 

The survey required active participation from the participants and involved completing a structured questionnaire. As the original questionnaire was in English and the target population spoke Chinese, the researcher conducted translations using the back-translation procedure [50]. This process ensures accurate and reliable translations by translating the questionnaire back to the original language version.

The questionnaire was divided into three sections. The first section collected data on the demographic traits of respondents, including age, gender, occupation, education, marital status, and income level. The second part of the questionnaire aimed to ascertain whether respondents had encountered or been exposed to mass media reports or communication-related topics such as natural disasters, environmental/climate matters, and pandemics. The third section of the questionnaire focused on measuring the correlations between various constructs of credibility, mass media exposure, social influence, perceptions, attitudes, and altruistic behavior by measuring them. This section aimed to determine the relationships and associations among these constructs, providing valuable insights into the study’s research objectives.

### 3.3. Measurement Items

The questionnaire design for the third section was primarily based on multiple-item measurement scales adapted from prior research on mass media exposure, credibility, social influence, perceptions, attitudes, and altruistic behavior. All measurement items were developed using 5-point rating scales with the following anchors: 1 = strongly disagree, 2 = disagree, 3 = neutral, 4 = agree, and 5 = strongly agree, except for the media exposure construct where the anchors were as follows; 1 = Never, 2 = Rarely, 3 = Occasionally, 4 = Often, 5 = Always. The research framework of this study consisted of six constructs, each with a designated number of items. Specific phrases were modified to better align with the scope of this research. Mass media exposure (ME) was defined as the attention or focus given to media outlets, such as television, newspapers, radio, and the Internet [18]. To measure this construct, a 5-point Likert scale was used to ask respondents how often they encounter mass media coverage regarding global crises and altruism from the four types of mass media: TV shows/news, newspapers, radio programs/news, and the web (α = 0.83). For this construct, the scale ranged from 1–5 where (1 = Never, 2 = Rarely, 3 = Occasionally, 4 = Often, 5 = Always) [12,18].

In this study, the credibility (PC) of a mass media source/message was assessed based on its ability to reflect reality and believability, using six measurement items [5]. Participants were asked to evaluate these items to determine their level of trust in the mass media’s reporting on critical world events, especially disaster-related news, and its validity (α = 0.81).

Social influence (SI) was measured using a four-item scale [7], where participants rated their agreement on a five-point Likert scale ranging from 1 (strongly disagree) to 5 (strongly agree). Sample items in this construct include “Unless I can get favorable reactions from others, I see no reason to use mass media” and “My private views about mass media news are different from those I express publicly” (α = 0.78). 

Public perceptions of mass media news (PU) were assessed using five items [7], previously used in another study [9]. Sample questions included “Mass media news provides accurate information about the needs of the public” and “Mass media news should be used to expose more public needs” (α = 0.84).

Attitudes toward mass media news were measured using a five-item scale [51], with items modified to suit the context of this study. Examples included statements like “It is a good and smart idea to use mass media platforms” and “Overall, my attitude towards mass media usage is favorable/positive” (α = 0.83).

Altruistic behavior/altruism was defined as intentional and voluntary behavior benefiting others without expecting external rewards, compensations, or avoiding punishment. A simplified 9-item SRA scale was used to measure altruism, as the original 20-item composition would be challenging to incorporate in the same questionnaire with other constructs [52]. Sample items included “I have ever given money to a charity organization” and “I have given goods or clothes to a charity as a result of the influence of news in the media” (α = 0.82). Negatively worded items were reverse coded before conducting reliability and validity tests and factor analysis on the scale items. This ensured that high values indicated the same type of response across all items. All data analyses were conducted in SPSS.

### 3.4. Data Analysis

Structural equation modeling (SEM) was the primary analytical tool used to test the hypotheses in this study. The Analysis of Moment Structures (AMOS) software, specifically the Covariance-Based Structural Equation Modeling (CB-SEM) approach, was employed for the analysis. CB-SEM is particularly suitable for sample sizes greater than 50 and provides more precise estimates in such cases [53]. The choice of CB-SEM over Variance-Based Structural Equation Modeling (VB-SEM) was based on the nature of the study, considering the adequate sample size and normally distributed data. CB-SEM is a parametric approach that requires normally distributed data. In contrast, VB-SEM is a non-parametric procedure suitable for smaller sample sizes and does not require the assumption of normal distribution [54]. 

Additionally, CB-SEM was chosen for its confirmatory nature, making it suitable for testing predetermined hypotheses, unlike VB-SEM, which is more exploratory [55]. To analyze the mediation effect, bootstrapping macro-PROCESS V4.2 in the IBM Statistical Package for Social Science (SPSS) version 22 was used. Bootstrapping is a resampling technique that estimates indirect effects and mediation in the model, providing more robust results and accounting for potential bias in the mediation effect analysis.

### 3.5. Descriptive Statistics of Sample Structure

The sample structure of the participants was analyzed by examining various demographic characteristics, which also served as control variables in the study. The demographic items and control variables included gender, age, income, and education qualification. Regarding gender, participants were asked to state their gender by indicating 1 = Male or 2 = Female. Out of the 435 remaining responses, 198 participants identified as males, representing 45.5%, while 237 participants identified as females, representing 54.5%. In terms of age, participants were given the option to select from predefined age ranges. The age range of 18–25 had the highest frequency, with 286 participants, accounting for 65.7% of the sample

On the other hand, the age range of “43 to 50” had the lowest frequency, with only 18 participants, representing 4.1%. Monthly income was measured by asking participants to indicate the range to which their monthly earnings belong. The majority of respondents (40.2%) reported a monthly income below TWD 30,000, while only a small percentage (5.7%) reported an income between TWD 50,001 and TWD 60,000. In terms of education qualification, 87.6% of the respondents possessed a bachelor’s degree, making it the most prevalent level of education in the sample. Participants with a doctorate (PhD.) had the lowest response rate, with only 0.2% of the sample reporting this qualification. These descriptive statistics provide insights into the demographic composition of the sample, which can be used as control variables in the analysis to understand how different factors influence the study’s variables.

### 3.6. Multicollinearity Assessment

The Tolerance and Variance Inflation Factor (VIF) was conducted to assess multicollinearity issues, which can be a concern if the VIF value exceeds 10 [55]. The results of the multicollinearity analysis in this study indicated that all the collected constructs had VIF values of less than 10, signifying the absence of significant multicollinearity issues. The highest VIF value observed in the study was 2.390, well below the threshold of 10. Thus, the research was not affected by multicollinearity problems, ensuring the reliability of the study’s findings.

### 3.7. Common Method Bias

Harman’s single-factor test was conducted to rule out or check for common method bias (CMB). The test involves examining whether the variance extracted for the first factor is lower than 50%. If the explained variance of the first factor is less than 50%, it suggests that CMB is not a significant concern in the research [56]. In this study, 33 elements were extracted, and the first factor accounted for 27.117% of the explained variance. This result indicates no severe problem with common method bias in the dataset, as the explained variance of the first factor was below the 50% threshold. Therefore, the study’s findings are not likely substantially affected by common method bias.

## 4. Results

### 4.1. Measurement Model Assessment

#### 4.1.1. Confirmatory Factor Analysis (CFA)

To assess the validity and reliability of the measures for the six constructs, a Confirmatory Factor Analysis (CFA) was conducted using Covariance Based Structural Equation Modelling (CB-SEM) in AMOS 21.0. The CFA was performed to evaluate the goodness of fit of the six-factor model before conducting hypothesis tests. The results of the CFA demonstrated a satisfactory level of fit for the model. The χ2 value was 943.51, with 359 degrees of freedom and a significance level of less than 0.001. Additionally, the Comparative Fit Index (CFI) value was 0.90, the Incremental Fit Index (IFI) value was 0.90, and the Root Mean Square Error of Approximation (RMSEA) value was 0.06, indicating a reasonable fitness level. Previous research has suggested that an RMSEA score below 0.05 signifies a “good fit,” while a score below 0.08 suggests a satisfactory match between the model and data [57]. Therefore, an RMSEA value of 0.06 indicates a good correspondence between the model and the data in our study.

These outcomes suggest that the participants were able to differentiate between the concepts being analyzed, as indicated in Table 1 below. The CFA results provide support for the validity and reliability of the measures used in the study, ensuring the robustness of the subsequent hypothesis testing.

#### 4.1.2. Composite Reliability, Discriminant, and Convergent Validity

Table 2 displays the Composite Reliability (CR) values for all constructs, ranging from 0.77 to 0.86, which indicates a high level of reliability. A CR value exceeding 0.7 ensures the reliability of all constructs [55], and our results demonstrate that all constructs meet this criterion. The Average Variance Extracted (AVE) was also calculated to assess the convergent validity of the model’s constructs. AVE measures the extent to which a construct accounts for variance compared to the variance resulting from measurement inaccuracies. AVE values higher than 0.5 are considered satisfactory for evaluating the convergent validity of the model’s constructs [55]. In some fields, AVE values higher than 0.4 may also be considered satisfactory [1].

The test results in Table 2 show that most AVE values exceed the threshold of 0.5, indicating strong convergent validity for the majority of the constructs. However, a few values show a slight decrease below 0.5, reaching 0.45. It is worth noting that AVE may be a conservative estimate of the measurement model’s validity [54,55]. Some researchers argue that based on composite reliability alone, the convergent validity of a construct can be considered satisfactory even if more than 50% of the variance is attributed to error. Although a few AVE values fall slightly below the recommended level of 0.5, the composite reliability of all constructs is notably higher than the recommended level, providing further support for the reliability and validity of the measurement items.

The high CR values and satisfactory AVE values in Table 2 indicate that the constructs used in the study are reliable and have strong convergent validity. These findings enhance the credibility of the study’s measurement model and support the subsequent hypothesis testing.

### 4.2. Examination of Research Hypotheses

The study utilized structural equation modeling to examine the research hypotheses and investigate the relationships among the constructs. The analysis results provide insights into the expected strength and significance of the hypothesized linkages between the variables, either confirming or rejecting the research hypotheses. Path coefficients in structural equation modeling are rated on a scale from negative one to positive one. A value close to +1 indicates a strong positive correlation, while a value relative to −1 indicates a strong negative correlation.

As shown in Table 3 and Table 4, all the relationships, except one (PU to AT), were positively related, with positive coefficient values. The results indicate that media exposure, perceived credibility, and social influence were significantly and positively associated with the public’s perceptions of mass media news, with coefficient values of 0.48 and 0.27 at *p* < 0.001 and 0.11 at *p* < 0.01, respectively. Therefore, these results provide supported hypotheses H1, H2, and H3.

Regarding the influence of peoples’ perceptions of mass media news on altruistic behavior (H4), the study found that perceptions about mass media news significantly and positively affect altruistic behavior (coefficient of 0.21, *SE* = 0.04 and *p* < 0.001). Therefore, hypothesis 4 was supported, indicating a direct relationship between perceptions about mass media news and altruistic behavior.

However, hypothesis 5, which suggested that perceptions about mass media news would influence people’s attitudes, was not supported. The results showed a negative relationship (coefficient of −0.18, *SE* = 0.06, and *p* < 0.003), which was inconsistent with the proposed hypothesis. To further investigate the relationship between perceptions about mass media news, attitudes, and altruistic behavior, the researchers conducted a bootstrap analysis to test hypothesis 6, which proposed the mediation effect of attitudes on the relationship between perceptions about mass media news and altruistic behavior. The results showed that the mediation effect was significant (estimate = −0.13, 95% CI [−0.20, −0.06]), supporting the existence of mediation.

Similarly, as hypothesized, people’s positive attitudes toward mass media news/messages were significantly and positively related to their altruistic behavior, with coefficients of 0.71, *SE* = 0.03 at *p* < 0.001. Thus, hypothesis 7 was supported, indicating a positive relationship between positive attitudes toward mass media news and altruistic behavior. Overall, the study provides evidence for the significant impact of perceptions about mass media news on altruistic behavior and the mediating role of attitudes in this relationship. The findings contribute to understanding the role of mass media in shaping individuals’ altruistic actions and responses to global crises.

**Figure 2:**
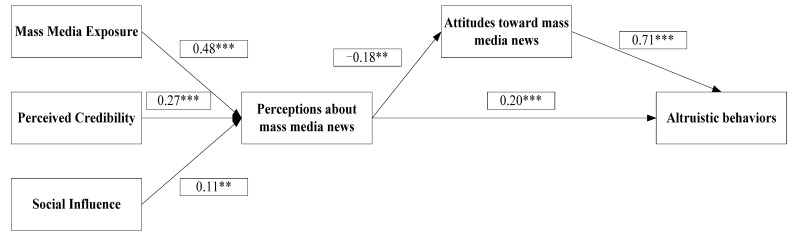
Standardized path estimates for the structural equation model. Note: N = 435; ** *p* < 0.01, *** *p* < 0.001.

Figure 2 represents the standardized path estimates for the structural equation model (SEM) used to test the proposed research framework. The standardized path estimates represent the strength and direction of the relationships between the different constructs in the model. The figure includes the standardized coefficients for each path, which indicate the strength of the relationship in terms of standard deviations. The figure also consists of the significance level (*p*-value) of each path estimate.

## 5. Discussion

Drawing on the cultivation theory and the TPB [9,12], this study aimed to investigate the factors that influence the public’s perceptions of the usage of mass media news regarding natural disasters, pandemics, and environmental issues. Further, the study aimed to examine the direct and indirect effects of these perceptions on public attitudes and altruistic behavior. The findings of the study have both theoretical and practical implications, which are discussed in this section.

The research findings highlighted several factors influencing the public’s perceptions of mass media news. People are increasingly turning to mass media, particularly TV and radio, as a primary and crucial source of information on emerging issues [58]. The media has the potential to disseminate knowledge, raise awareness, and shape individuals’ attitudes and behaviors [58]. As the media extensively covers emerging topics, it can significantly impact people’s understanding and opinions, ultimately influencing their responses to crises and emergencies [20]. This study examined how mass media coverage affects the public’s perceptions and behaviors, recognizing the importance of media information during critical events, such as natural disasters. 

During crises, individuals heavily rely on media coverage to make sense of their surroundings and stay informed about ongoing events. The mass media gradually influences people’s behavior, emotions, and thoughts in response to the information they receive [58]. The study’s findings contribute to a better understanding of how media messages can shape public perceptions and encourage altruistic behaviors, particularly in the context of crisis relief initiatives and charitable actions. This research underscores the importance of crafting media messages with care, especially during emergencies and global crises. Mass media can mobilize public support and engagement in response to pressing issues by conveying information and promoting altruism. Policymakers, organizations, and media practitioners can leverage the power of mass media to create public awareness, foster positive attitudes, and encourage collective efforts toward social good.

The results of this study support the proposed hypothesis that mass media exposure, the credibility of the news, and social influence significantly influence people’s perceptions of mass media usefulness, which, in turn, affect their attitudes and behaviors. These findings are consistent with previous research, which has also shown a positive correlation between exposure to mass media and the public’s perceptions of its usefulness. For instance, previous studies have found that individuals’ perceptions of disasters are significantly influenced by their exposure to mass media, with television being the most impactful and widely used medium [9]. This highlights the significant role that mass media plays in shaping how people perceive and interpret social realities, particularly regarding disaster-related news.

The cultivation theory, which posits that frequent exposure to mass media shapes individuals’ views of social realities, is supported by the findings of this study. The more individuals are exposed to mass media, the more their perceptions of the relevance and usefulness of the conveyed news or messages are influenced [9]. This is also reflected in other research on environmental issues, which found that mass media has a significant impact on the public’s perspectives [20]. Likewise, the study indicates that exposure to mass media has a stronger effect on the public’s perception of the usefulness of the news/message compared to the perceived credibility and social influence of the media, as evidenced by the higher coefficient value of 0.48 compared to 0.27 and 0.11 for credibility and social influence, respectively.

As expected, the study found a direct relationship between individuals’ perceptions of mass media credibility and their perceptions of its usefulness. When people trust the news or media sources they use, they are more likely to engage with and understand the information presented. This leads them to recognize the value of the message and its source, reducing their need to seek information from alternative sources. To build and maintain the confidence of their audience, media organizations should employ effective methods of disseminating information. One research emphasized the significance of audience trust in the media, underscoring the importance of ensuring that media organizations are viewed as trustworthy by their viewers [5]. Maintaining trust is crucial for media organizations as it influences the public’s perception of the usefulness and reliability of the information they provide. When individuals have confidence in the media, they are more likely to rely on it as a credible source of information, which can lead to positive attitudes and behaviors, such as engaging in altruistic activities and supporting charitable causes.

This study also revealed a significant and positive correlation between social influence and individuals’ perceptions of the effectiveness of mass media. This contrasts with previous studies, which demonstrated a negative impact of social influence on perceived usefulness [7]. One of the previous studies found no correlation between perceived use and social influence [30]. The disparity in findings can be attributed to the different settings of our study compared to the research focused on young clients in the banking industry [7]. The present study observed that social influence plays a crucial role in shaping individuals’ perceptions of mass media news. This aligns with the notion that people often orient their behavior based on social comparison and exchange, with socialization agents serving as important sources of observing and learning values, norms, attitudes, and behaviors. Among these socialization agents, mass media stands out as a significant influencer. Therefore, social influence indeed has an impact on how individuals perceive mass media news, subsequently influencing various behaviors.

The confirmation of hypothesis 4 affirms that people’s positive perceptions of mass media news regarding altruism directly impact their altruistic behavior. Mass media news has the power to foster optimistic attitudes toward generosity and assistance among individuals. Those who perceive the mass media’s crisis coverage as beneficial are more likely to be driven to help those in need and engage in altruistic behaviors. This finding aligns with the TBP, which asserts that a prior intention to act is crucial before engaging in a behavior. Therefore, people’s perceptions of behavior, collective expectations, and beliefs in its benefits influence their involvement in that behavior. In this case, positive perceptions of mass media news can encourage individuals to engage in acts of altruism.

However, the hypothesis that people’s attitudes toward mass media would be positively related to their perceptions of mass media news was disproven, as there was a negative relationship between the two variables. This means that the fifth hypothesis cannot be supported. This discovery contradicts previous investigations, which found a positive association between perceptions and attitudes [8]. Additionally, another study established that the most significant factors shaping one’s attitude are the evaluations of specific effects or perceptions [39]. In light of this negative relationship, it is suggested that personalized approaches should address individuals’ negative perceptions of mass media news rather than relying solely on mass communication [6].

The negative relationship between perceptions about mass media news and attitudes toward mass media news can be attributed to the age of the respondents, with the majority falling within the 18 to 25 age range. Young individuals tend to develop negative perceptions and attitudes towards traditional mass media and may prefer social media platforms. This preference for social media could lead to a perception of mass media news as untrustworthy, which can influence their media usage and behavioral responses. Additionally, the public might only trust certain information from mass media news during a crisis, leading to biased perceptions and negative attitudes toward the news [59].

On the other hand, the study has confirmed the existence of the mediation effect by attitudes toward mass media news in the relationship between public perceptions of mass media news and altruistic behavior, supporting hypothesis 6. This implies that individuals’ perceptions can influence their altruistic behavior through their attitudes toward mass media news. The negative mediation effect aligns with the negative relationship found in hypothesis 5. To encourage positive attitudes and promote altruistic behavior, mass media entities should focus on attracting the attention of young adults when disseminating news about altruism. This will help shape their future behavior and increase acts of altruism. Furthermore, during times of crisis, it is crucial for mass media entities to provide accurate and trustworthy information to ensure that the public can develop positive attitudes toward the news and be motivated to engage in altruistic behavior.

As hypothesized, attitudes toward mass media news positively influenced altruistic behavior. This implies that individuals with positive attitudes toward mass media news are more likely to engage in giving and helping behavior, while those with negative attitudes are less likely to do so due to perceptions of unreliability or lack of legitimacy. This finding is consistent with the TPB, which posits that people’s behavior is influenced by their attitudes, which is defined as how they view and evaluate an object [39]. According to the TPB, attitudes play a significant role in predicting people’s actions.

Previous research has also suggested that individuals with favorable views of mass media news/messages related to altruism are more inclined to participate in altruistic behaviors [40]. Therefore, this study provides further evidence to support the TPB’s argument that the public’s attitudes toward news and information disseminated by mass media play a crucial role in determining their level of involvement in altruistic activities. By understanding and shaping these attitudes, mass media entities can potentially promote and encourage more altruistic behavior among the audience.

## 6. Theoretical and Practical Implications

The research has made a noteworthy contribution to the study of altruism. This study’s significance can be demonstrated in both theoretical and managerial aspects. Theoretically, the study has contributed to the current body of literature by identifying the factors that can affect a person’s inclination toward altruistic actions. Previous research studies primarily concentrated on consumer behavior, with little attention paid to the humanitarian aspect, as evidenced by the limited studies on perceptions and attitudes.

The study’s contribution is significant both in theoretical and managerial aspects. Theoretically, this research adds to the existing literature by identifying factors influencing individuals’ inclination toward altruistic actions. Previous studies have primarily focused on consumer behavior, giving limited attention to the humanitarian aspect, especially perceptions and attitudes. By examining the relationships between different factors and their impact on perceptions, attitudes, and altruistic behavior, this study provides valuable insights into the role of mass media in disseminating information about emerging crises. It highlights how mass media influences people’s behavior, emotions, and thoughts during humanitarian efforts. Moreover, the study supports and extends the arguments related to the cultivation theory and the theory of planned behavior, adding evidence to these theoretical frameworks.

From a managerial perspective, the findings of this research can guide mass media entities in shaping their content to promote altruistic behavior among the audience. Understanding the factors that influence perceptions and attitudes can help media organizations create more effective messages that resonate with the public and encourage them to engage in acts of altruism. By leveraging the potential of mass media to disseminate information about humanitarian causes, media entities can play a significant role in fostering a more altruistic and compassionate society.

The example of the Da Ai TV Station of the Tzu Chi Foundation in Taiwan serves as a compelling illustration of the powerful influence that mass media can have on shaping public perceptions and inspiring altruistic behavior. Da Ai TV Station’s focus on broadcasting programs and news centered around good deeds and helping others has a profound impact on viewers’ perceptions and attitudes. Through its constant coverage of positive and altruistic actions, the TV station helps purify and change the perceptions of people in society. By consistently highlighting stories of kindness, compassion, and charitable activities, Da Ai TV fosters a sense of empathy and encourages viewers to embrace altruism as a core value. This media influence extends beyond mere perception changes: it motivates people to act and get involved in charitable activities. The station’s emphasis on promoting goodness and helping others inspires many volunteers to join the Tzu Chi Foundation, contributing their time, money, and efforts to support various charitable initiatives. The example of Da Ai TV demonstrates how a media platform focusing on promoting altruism and good deeds can have a transformative effect on public perceptions and behaviors. By leveraging the power of mass media for positive social change, organizations like Tzu Chi Foundation exemplify how media can inspire collective action and create a more compassionate and caring society.

The findings of this research emphasize the importance of reliable and trustworthy mass media news in promoting altruistic behavior. People’s perceptions and attitudes toward the news can significantly influence their willingness to engage in selfless actions. Therefore, mass media organizations should be mindful of their impact on their audience and aim to create positive views and mindsets toward humanitarian news. To achieve this, media entities should focus on delivering accurate and reliable information and employing effective strategies to gain the trust of their audience. Allocating more resources to mass media and thoroughly verifying sources can help enhance the credibility of the information disseminated, increasing the public’s confidence in the news. 

Furthermore, media organizations should be cautious about the potential negative impact of exposure to negative messages. Negative perceptions of news may discourage individuals from participating in altruistic behavior. Therefore, media entities should strive to balance reporting on critical issues and providing positive, uplifting content that inspires altruistic actions. This study highlights the pivotal role that mass media can play in shaping public attitudes and behaviors toward humanitarian efforts. By leveraging this influence responsibly, media entities can contribute to building a more compassionate and altruistic society, fostering a collective willingness to provide aid and support during times of crisis.

Indeed, regular public education and dissemination of information through reliable media channels are necessary to foster people’s altruistic behaviors, especially in the face of emerging issues and crises. Media plays a crucial role in influencing public attitudes and actions, and as such, it should provide accurate and trustworthy information to encourage altruism among the audience. To achieve this, media entities must offer comprehensive reporting on various emerging topics, ensuring that the information is timely, accurate, and easily accessible to the public. This includes providing detailed coverage before, during, and after crises, allowing for people to understand the reasons, foreseeability, and consequences of emergencies.

Furthermore, delivering information in the native language is vital for effective communication and comprehension. This approach ensures that the message is accessible to a broader audience and fosters a deeper understanding of the issues at hand. By adhering to these principles, media organizations can effectively contribute to shaping positive perceptions of communication channels and promoting altruistic behaviors in society. As a result, people will be more inclined to engage in acts of kindness and humanitarian efforts, creating a compassionate and empathetic community that responds effectively to various challenges and crises.

## 7. Limitations and Future Research Directions

Like any other study, this study was not spared from having some limitations. One of the limitations that can be pointed out in this study is the generalizability of the findings as far as the sample size is concerned. The study’s sample consisted of only those with access to mass media (TV, radio, and newspapers) and those who had experienced social influence. Furthermore, while this study relied on a scientific sampling procedure, the sample cannot completely represent the general population in Taiwan. Our model was selective in nature, collecting data from Eastern Taiwan. Though diverse, this sample was not based on a nationwide sampling frame. The findings of this study were not generalizable to the entire population in Taiwan. Future studies may consider including all regions in Taiwan.

Indeed, the generalizability of the study’s findings is a valid limitation due to the sample size and its selective nature. The study’s reliance on participants with access to mass media and exposure to social influence may limit the applicability of the results to the broader population in Taiwan. While the study employed a scientific sampling procedure, the sample was restricted to Eastern Taiwan, which may only partially represent part of the population of the country. To improve the generalizability of future research, it is essential to consider including participants from diverse regions across Taiwan. A nationwide sampling frame would allow for a more comprehensive representation of the population, enhancing the external validity of the study’s findings.

Secondly, future research could redesign the model by linking factors affecting perceptions to altruistic behavior, potentially yielding different results, such as media exposure directly influencing people’s engagement in humanitarian acts. Similarly, the model could introduce moderators to gauge whether the relationship between perceptions/attitudes and altruistic behavior can be strengthened or weakened under certain circumstances. The study used a cross-sectional approach to collect data using a survey conducted only once; hence, it might have posed standard method bias issues. Future studies must consider collecting data using two-time lagged surveys or employing other designs like the longitudinal approach. Future research may also consider investigating media consumption by age and how this influences media users’ perceptions, attitudes, and altruistic behavior. This will allow for researchers to uncover which age group primarily engages in altruistic behavior.

## 8. Conclusions

The findings of the present study provide further support to previous research, indicating that media exposure plays a significant role in shaping people’s perceptions of mass media news, especially on various emerging topics. Furthermore, the study highlights the importance of credibility and social influence in shaping how individuals perceive mass media news. It is evident that attitudes toward mass media news have a substantial impact on individuals’ inclinations to engage in altruistic behavior. Moreover, the study reveals that attitudes toward mass media news mediate the relationship between public perceptions and altruistic behavior. However, contrary to our expectation, the public perception of mass media news was negatively related to attitudes towards mass media news, which contrasts with findings from previous studies.

The present study expands the application of behavioral theories in the context of altruism by incorporating the cultivation theory and the theory of planned behavior. Theoretically, this research contributes to the existing literature by investigating factors that influence the perception of mass media news, ultimately influencing altruistic behavior indirectly. Furthermore, the study adds value to the existing literature by emphasizing the role of mass media in disseminating information regarding emerging crises, which, in turn, influences people’s behavior, emotions, and thoughts. By doing so, this study provides valuable insights to mass media organizations, highlighting the importance of communicating reliable and trustworthy news to promote acts of altruism. This emphasis on responsible news reporting aims to foster positive views and mindsets among the audience, leading to an increased willingness to engage in humanitarian acts during times of crisis.

## Figures and Tables

**Table 1:** Results of Confirmatory Factor Analysis for the Measures of Variables Studied.

Models	χ^2^	*df*	∆χ^2^	∆*df*	CFI	IF	RMSEA
Six-factor model	943.51	359	-	-	0.90	0.90	0.061
Five-factor model	1315.04	367	371.54 ***	8	0.84	0.84	0.08
Four-factor model	2374	371	1430.49 ***	12	0.67	0.67	0.11
Three-factor model	2793.65	374	1850.14 ***	15	0.60	0.60	0.12
Two-factor model	2846.45	376	1902.94 ***	17	0.59	0.59	0.12

*** *p* < 0.001. Note: CFI = comparative fit index; IFI = Incremental Fit Index; RMSEA = root-mean-square error of approximation. Six-factor model: media exposure, credibility, social influence, perceptions, attitudes, and altruistic behavior. Five-factor model: media exposure and credibility combined. Four-factor model: perceptions and attitudes combined. Three-factor model: media exposure, credibility, and social influence combined. Two-factor model: perception, attitude, and altruistic behavior combined.

**Table 2:** Means, standard deviations, correlations, and reliability.

Variables	Mean	SD	CR	AVE	1	2	3	4	5	6
1. Media exposure (ME)	3.50	0.98	0.83	0.56	(0.75)					
2. Credibility (PC)	3.87	0.64	0.84	0.51	0.52 **	(0.71)				
3. Social influence (SI)	3.14	0.90	0.77	0.46	0.12 *	−0.07	(0.68)			
4. Perception (PU)	4.48	0.61	0.83	0.56	0.21 **	0.12 *	0.10 *	(0.75)		
5. Attitudes (AT)	4.12	0.79	0.84	0.52	0.54 **	0.65 **	−0.07	−0.04	(0.72)	
6. Altruistic behavior (AB)	3.78	0.73	0.86	0.48	0.50 **	0.58 **	−0.042	0.11 *	0.48 **	(0.69)

Note: N = 435; * *p* < 0.05; ** *p* < 0.01 reliability coefficients are reported along the diagonal parentheses.

**Table 3:** Examination Results of Research Hypotheses.

	Estimate	S.E.	C.R.	P	Remarks
ME→PU	0.48	0.05	9.79	***	H1: Supported
PC→PU	0.27	0.05	5.31	***	H2: Supported
SI→PU	0.11	0.04	2.78	0.006	H3: Supported
PU→AB	0.21	0.04	5.37	***	H4: Supported
PU→AT	−0.18	0.06	−2.10	0.003	H5: Not supported
AT→AB	0.71	0.03	23.28	***	H7: Supported

Note: *** *p* < 0.001 ME = Media exposure, PC = Credibility, SI = Social Influence, PU = Perception, AT = Attitudes, AB = Altruistic Behavior.

**Table 4:** Bootstrapping Results for Indirect Effects.

Rival Path	Estimated effect	Bootstrapping 95% CI
		Bias-Corrected (LL, UL)
PU-AT-AB	0.13	(−0.20, −0.06)

Note: PU = Perception of the public; AT = Attitudes; AB = Altruistic behavior.

## Data Availability

Data sharing is not applicable.
